# Triamcinolone acetonide activates an anti-inflammatory and folate receptor–positive macrophage that prevents osteophytosis in vivo

**DOI:** 10.1186/s13075-015-0865-1

**Published:** 2015-12-05

**Authors:** Michiel Siebelt, Nicoline Korthagen, Wu Wei, Harald Groen, Yvonne Bastiaansen-Jenniskens, Christina Müller, Jan Hendrik Waarsing, Marion de Jong, Harrie Weinans

**Affiliations:** Department of Orthopaedics, Erasmus Medical Centre, P.O. Box 2040, 3000 CA Rotterdam, The Netherlands; Department of Otorhinolaryngology, Erasmus Medical Centre, Rotterdam, The Netherlands; Department Orthopaedics, UMC Utrecht, Utrecht, The Netherlands; Department Rheumatology, UMC Utrecht, Utrecht, The Netherlands; Department of Nuclear Medicine, Erasmus Medical Centre, Rotterdam, The Netherlands; Centre for Radiopharmaceutical Sciences, Swiss Federal Institute of Technology (ETH) Zurich, Paul Scherrer Institute, University Hospital Zurich, Villigen, Switzerland; Department of Radiology, Erasmus Medical Centre, Rotterdam, The Netherlands; Department of Biomechanical Engineering, Delft University of Technology, Delft, The Netherlands

**Keywords:** Osteoarthritis, Triamcinolone acetonide, Macrophage activation, Cartilage degradation, Subchondral bone

## Abstract

**Introduction:**

Triamcinolone acetonide (TA) is used for osteoarthritis management to reduce pain, and pre-clinical studies have shown that TA limits osteophyte formation. Osteophyte formation is known to be facilitated by synovial macrophage activation. TA injections might influence macrophage activation and subsequently reduce osteophytosis. Although widely applied in clinical care, the mechanism through which TA exerts this effect remains unknown. In this animal study, we investigated the in vivo effects of TA injections on macrophage activation, osteophyte development and joint degeneration. Furthermore, in vitro macrophage differentiation experiments were conducted to further explain working mechanisms of TA effects found in vivo.

**Methods:**

Osteoarthritis was induced in rat knees using papain injections and a running protocol. Untreated and TA-treated animals were longitudinally monitored for 12 weeks with in vivo micro–computed tomography (μCT) to measure subchondral bone changes. Synovial macrophage activation was measured in vivo using folate receptor β (FRβ)-targeted single-photon emission computed tomography/computed tomography. Articular cartilage was analyzed at 6 and 12 weeks with ex vivo contrast-enhanced μCT and histology. To further explain the outcomes of our in vivo study, TA on macrophages was also studied in vitro. These cultured macrophages were either M1- or M2-activated, and they were analyzed using fluorescence-activated cell sorting for CD163 and FRβ expression as well as for messenger RNA (mRNA) expression of interleukin (IL)-10.

**Results:**

Our in vivo study showed that intra-articular injections with TA strongly enhanced FRβ^+^ macrophage activation. Despite stimulated macrophage activation, osteophyte formation was fully prevented. There was no beneficial effect of TA against cartilage degradation or subchondral bone sclerosis. In vitro macrophage cultures showed that TA strongly induced monocyte differentiation towards CD163^+^ and FRβ^+^ macrophages. Furthermore, TA-stimulated M2 macrophages showed enhanced IL-10 expression at the mRNA level.

**Conclusions:**

TA injections potently induce a CD163^+^- and FRβ^+^-activated macrophage with anti-inflammatory characteristics such as reduced IL-10 production in vitro and lack of osteophytosis in vivo.

## Introduction

Osteoarthritis (OA) is characterized by deterioration of articular cartilage and extensive subchondral bone remodelling [1, 2], as well as by inflammation within the synovial lining of the osteoarthritic joint [3]. During OA progression, synovial macrophages become activated and secrete many pro-inflammatory cytokines and growth factors. These cytokines and growth factors are thought to detrimentally change the articular joint.

First, activated synovial macrophages have been proposed to enhance transforming growth factor (TGF)-β production. Due to TGF-β, synoviocytes increase their production of bone morphogenetic protein 2 (BMP2) and BMP4; as a consequence, osteophytes develop within the OA joint [4, 5]. Second, it is thought that enhanced growth factor and cytokine production by activated macrophages facilitates cartilage extracellular matrix (ECM) degradation, contributes to synovial fibrosis [6] and induces pain [7]. The latter is of special interest because pain management plays a pivotal role in clinical management of OA.

Pain management for patients with OA can be achieved through analgesia with agents such as paracetamol, non-steroidal anti-inflammatory drugs or intra-articular injection of corticosteroids. Intra-articular injection with corticosteroids provides excellent results for OA-related pain [8] and is an advocated treatment for individuals with knee OA [9]. More specifically, triamcinolone acetonide (TA) injections are even more effective than other corticosteroids in reducing pain [10].

In 1985, Williams et al. reported that TA quite effectively protected against osteophyte development in a pre-clinical model of OA [11]. This finding suggests that TA somehow intervenes with synovial macrophage activation and might prevent subsequent TGF-β–induced osteophyte development. More recently, in 2014, this finding was reproduced in a post-traumatic model of OA using intra-articular injections of dexamethasone [12]. The authors of that study also showed that corticosteroid therapy reduced cartilage destruction. It remains unclear through which mechanisms corticosteroids exert this positive effect on macrophages and other joint tissues within the joint during OA development. This effect might result from the marked influence of corticosteroids on macrophage differentiation.

Inactive macrophages are able to differentiate into different active subtypes. First, the classically activated (or M1) macrophages are activated through a cell-mediated immune response. Interferon (IFN)-γ, lipopolysaccharides and tumour necrosis factor (TNF) are especially well-known inducers of M1 macrophages [13, 14]. Alternatively activated (M2) macrophages are related to humoral immunity tissue repair [15]. Interleukin (IL)-4 is known to induce a wound-healing, M2-activated macrophage whose activity is related to tissue repair [16]. Interestingly, in response to corticosteroids, yet another activated macrophage subtype develops; these are known as regulatory macrophages [17]. Regulatory macrophages are considered anti-inflammatory and produce large amounts of IL-10 [18]. Intra-articular injection of TA might polarize macrophage activation towards this specific form of M2 phenotype with subsequent beneficial effects on osteophyte formation and cartilage degradation.

Recently, we established an in vivo model of severe OA that shows severe degradation of articular cartilage, enhanced subchondral bone sclerosis formation and pronounced osteophyte formation [19]. Using folate receptor β (FRβ)-targeted single-photon emission tomography/computed tomography (SPECT/CT) to quantitatively measure macrophage activation [20, 21], we also found abundant activation of synovial macrophages within knee joints in this rat OA model [19]. In this rat model of severe OA, we investigated the in vivo effect of intra-articular TA injections on macrophage activation using FRβ-targeted SPECT/CT. We hypothesized that intra-articular treatment with TA reduces the amount of macrophage activation and therefore diminishes osteophyte formation as described by Williams et al. [11]. Furthermore, using longitudinally applied micro–computed tomography (μCT) for in vivo bone analysis and ex vivo equilibrium partitioning of an ionic contrast agent using micro–computed tomography (EPIC-μCT), we also analyzed whether intra-articular TA injections might have a beneficial effect on OA-related subchondral sclerosis and cartilage degradation as well. To explain our in vivo results, we performed several in vitro experiments. In these experiments, we characterized M1- and M2-differentiated macrophages by their cell surface receptor expression. We analyzed whether the addition of TA could polarize macrophages towards a certain subtype and whether TA influences FRβ expression.

## Materials and methods

### Effects of intra-articular injections of TA on severe osteoarthritis progression

Forty 16-week-old male Wistar rats (Charles River Netherlands, Maastricht, The Netherlands) were housed in the animal facility of the Erasmus Medical Centre under a 12-h light-dark regimen at 21 °C during the experimental period, and all animals received standard food pellets and water ad libitum. Animals were divided into two groups: 20 rats served as untreated OA controls, and another 20 rats were treated during the experiments with weekly intra-articular injections of TA. TA (Kenacort; Bristol-Myers Squibb, Woerden, The Netherlands) was diluted with saline to a concentration of 1.43 mg/ml. Animals were given weekly injections of 70 μl of this solution (with 100 μg of TA) via a 27-gauge needle (Sherwood-Davis & Geck, Gosport, UK) in their OA-induced knee joint. TA was chosen because of its superior function compared with another corticosteroid, betamethasone, in reducing pain in human patients [8].

**Fig. 1:**
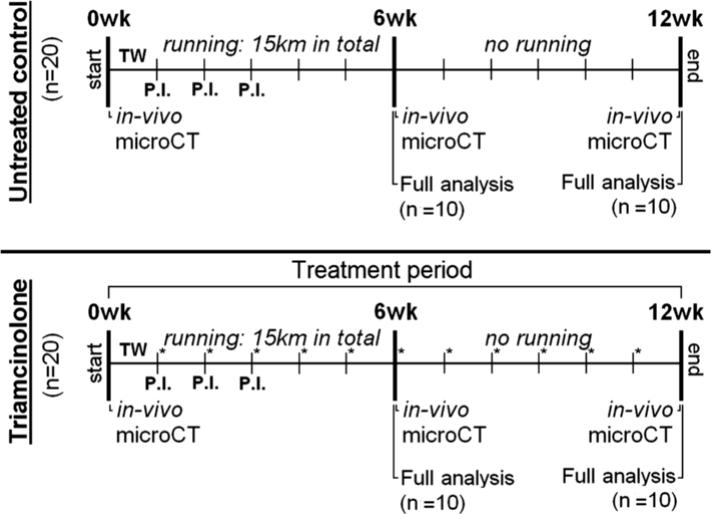
Experiment design indicating analytical time points and methods for each experimental group. Forty 16-week-old male Wistar rats were given three papain intra-articular injections (P.I.) and forced to run 15 km on a motorized treadmill. Animals were divided into two different groups: an untreated osteoarthritis (OA) group (*n* = 20) and a group treated with intra-articular triamcinolone acetonide (TA) injections (*n* = 20). TA-treated animals were treated with weekly intra-articular TA injections (100 μg/injection), indicated with *asterisks* in the scheme. During the experiment, three longitudinal micro–computed tomographic (microCT) scans were made to measure subchondral bone changes. At 6 and 12 weeks, a full-analysis sequence was done in ten animals per group, consisting of in vivo determination of activated macrophages using single-photon emission computed tomography/computed tomography and ex vivo cartilage analysis with equilibrium partitioning of an ionic contrast agent using micro-CT and histology.

In all animals in both experimental groups, severe OA was induced using intra-articular papain injections in the left knee joints, combined with exposure to a moderate exercise protocol as described previously [19]. In short, all animals received three intra-articular injections that consisted of 15 μl of 4 % (wt/vol) papain solution (type IV, double-crystallized, 15 U/mg; Sigma-Aldrich, St. Louis, MO, USA) with 15 μl of 0.03 M l-cysteine (Sigma-Aldrich) [22]. The contralateral knee joint served as an internal healthy control. All rats were forced to run on a motorized rodent treadmill (LE-8700; Panlab Harvard Apparatus, Barcelona, Spain) for 6 weeks, covering a distance of 15 km (500 m/day, 5 days/week) [19]. During the study, all animals were longitudinally monitored with μCT to measure subchondral bone changes. At 6 and 12 weeks, ten rats in both groups were selected for a full-analysis sequence. This sequence consisted of SPECT/CT to quantify in vivo macrophage activation [23] and ex vivo EPIC-μCT and histology to measure cartilage quality [24]. For all procedures, exactly the same procedures as described previously were followed [19]. The animal ethics committee of the Erasmus Medical Centre, Rotterdam, The Netherlands, approved all conducted procedures. A detailed planning scheme of all groups and conducted tests is given in Fig. 1.

### Subchondral bone measurements on μCT scans

Both knees of all animals were scanned by μCT while the animals were under isoflurane anaesthesia, using a SkyScan 1176 in vivo μCT scanner (Bruker microCT, Kontich, Belgium). Ten minutes of scan time per knee was required at an isotropic voxel size of 18 μm, a voltage of 65 kV, a current of 385 mA, and a field of view of 35 mm using a 1.0-mm aluminium filter over 198 degrees with a 0.5-degree rotation step and a 270-millisecond exposure time. All datasets were segmented with a local threshold algorithm [25]. Cortical and trabecular bone were automatically separated using in-house software [26]. Using SkyScan software, both subchondral plate thickness (in micrometres) and subchondral plate porosity (in cubic millimetres) of the medial and lateral compartment of the tibial plateau were measured [27]. In the tibial epiphysis, we measured the trabecular thickness (in micrometres) and trabecular bone volume fraction (BV/TV), representing the ratio of trabecular bone volume (in cubic millimetres) to endocortical tissue volume (in cubic millimetres). We also quantified the amount of ectopic bone formation as a measure of osteophyte growth (in cubic millimetres) on longitudinal μCT scans.

### Determination of activated macrophages by SPECT/CT using ^111^In-EC0800

Activated macrophages express FRβ, which allows monitoring of macrophages in vivo using folate-based radiotracers [20, 21, 28]. Phosphate-buffered saline (pH 6.5) with DOTA-Bz-folate (DOTA-Bz-Folate, EC0800; kindly provided by Endocyte, West Lafayette, IN, USA) [1, 29] was labelled with ^111^InCl_3_ (Covidien, Petten, The Netherlands) as described previously [19]. Quality control was performed by instant thin-layer chromatography with a silica gel, which revealed a radiochemical yield of approximately 91 % at a specific activity of 50 MBq/μg. ^111^In-EC0800 (55 MBq) was administered via the tail vein 20 h before scanning. SPECT/CT scans were obtained with a four-head multiplex multi-pinhole small-animal SPECT/CT camera (NanoSPECT/CT^TM^; Bioscan, Washington, DC, USA). All knee joints were scanned with both helical μCT (acquisition time 5 minutes) and SPECT (acquisition time 30 minutes). All scans were analyzed using InVivoScope processing software (Mediso, Boston, MA, USA). To reduce inter-individual variation, the absolute difference in measured radioactivity (kilobecquerels per cubic millimetre) of the OA knee joint compared with the internal control joint was calculated. This absolute difference was used when we compared means of untreated animals with those of TA-treated animals.

### Cartilage evaluation with contrast-enhanced μCT and histology

EPIC-μCT has a strong correlation with cartilage sulphated glycosaminoglycan (sGAG) content [24]. The animals were killed directly after the last SPECT/CT scan, and both knee joints were harvested for EPIC-μCT analysis. All specimens were incubated in 40 % solution of ioxaglate for 24 h at room temperature [30]. EPIC-μCT was performed with the same μCT scanner using the following scan settings: an isotropic voxel size of 18 μm, a voltage of 65 kV, a current of 385 mA, a field of view 35 mm, a 0.5-mm aluminium filter, 198 degrees with a 0.5-degree rotation step, and a 235-millisecond exposure time. In all EPIC-μCT datasets, X-ray attenuation (arbitrary grey values related to sGAG content) and cartilage thickness (in micrometres) were calculated separately for cartilage of the medial and lateral plateaus of the tibia [19].

After EPIC-μCT, the separated parts of the knee joints were fixed in paraformaldehyde, decalcified with formic acid and embedded in paraffin. Sagittal sections were made at 300-μm intervals and stained with Safranin-O to scan the amount and distribution of the glycosaminoglycans. Sections were stained all at once to minimize artefacts between different samples.

### Surface receptor expression on monocyte-derived macrophages in vitro

Monocytes were isolated from peripheral blood of healthy human donors using sequential Ficoll-Hypaque and Percoll density gradients (GE Healthcare, Uppsala, Sweden) and cultured in GIBCO RPMI/GlutaMAX medium (Life Technologies, Merelbeke, Belgium) with addition of penicillin (100 U/ml), streptomycin (100 μg/ml) and 10 % foetal calf serum (Life Technologies). Monocyte-derived macrophages were generated by culturing monocytes for 7 days in the presence of 800 U/ml human recombinant granulocyte-macrophage colony-stimulating factor (GM-CSF; for M1 subtype differentiation) or 25 ng/ml human recombinant macrophage colony-stimulating factor (M-CSF; for M2 subtype differentiation) (GM-CSF and M-CSF were both acquired from R&D Systems, Minneapolis, MN, USA). To study the influence of TA on macrophage differentiation, 100 nM TA (Kenacort) was added to the culture medium during these 7 days. The culture medium was refreshed after 3–4 days.

### Flow cytometry

The expression of membrane receptors was evaluated by incubating the cells with specific fluorescent antibodies. First, the cells were incubated with a rabbit anti-human folate receptor 2 antibody (Thermo Fisher Scientific, Rockford, IL, USA) at 4 °C for 30 minutes in the presence of rabbit serum. This step was followed by incubation with a fluorescein isothiocyanate–labelled goat anti-rabbit antibody (Thermo Fisher Scientific), CD80-phycoerythrin (clone L307.4; BD Biosciences, San Jose, CA, USA), CD163-PerCP-cyanine 5.5 (clone GHI/61; BioLegend, San Diego, CA, USA), CD14 allophycocyanin (APC)-AF750 (clone RMO52; Beckman Coulter, Brea, CA, USA), CD206-PC7 (clone 3.29B1.10; Beckman Coulter) and CD16-APC (clone 3G8; Life Technologies, Frederick, MD, USA). Flow cytometry was performed on a FACSCanto II cytometer (BD Biosciences) according to the manufacturer’s protocols. Fluorescence minus one controls were used to identify gating boundaries. Values were expressed as mean fluorescence intensity ratio compared with an unstained control (fold change).

### Detection of IL-10 mRNA levels by real-time quantitative PCR

Messenger RNA (mRNA) was isolated using an RNeasy Mini Kit (QIAGEN, Venlo, The Netherlands). After on-column DNase I treatment (RNase-Free DNase kit; QIAGEN), RNA was quantified using a NanoDrop ND-1000 spectrophotometer (NanoDrop/Thermo Scientific, Wilmington, DE, USA) and reverse-transcribed into complementary DNA using the iScript cDNA Synthesis Kit (Bio-Rad Laboratories, Veenendaal, The Netherlands). Gene expression was analyzed using the CFX384 Real-Time PCR Detection System (Bio-Rad Laboratories). The quantitative polymerase chain reactions were performed in duplicate in 384-well plates in a final volume of 10 μl using IQ SYBR Green Supermix (Bio-Rad Laboratories). *IL-10* mRNA levels were normalized to those of the reference genes, *TBP* and *HPRT*. The primers used were as follows: IL-10 forward 5′-GACTTTAAGGGTTACCTGGGTTG-3′, reverse 5′-TCACATGCGCCTTGATGTCTG-3′; TBP forward 5′-TGCACAGGAGCCAAGAGTGAA-3′, reverse 5′-CACATCACAGCTCCCCACCA-3′; and HPRT forward 5′-TATTGTAATGACCAGTCAACAG-3′, reverse 5′-GGTCCTTTTCACCAGCAAG-3′.

### Statistical analysis

For the in vivo study, differences between means of OA-induced and healthy knee joints within the same animal were tested using paired *t* tests at each time point for all outcome parameters (GraphPad Software, La Jolla, CA, USA). When we compared differences between means of untreated OA animals and TA-treated animals, an unpaired *t* test was used at each time point for all outcome parameters (GraphPad Software). Statistical significance among the different cell treatments was assessed using one-way analysis of variance with Bonferroni’s correction (IBM SPSS software; IBM, Armonk, NY, USA). Longitudinal data from in vivo μCT were additionally analyzed using generalized estimating equations (GEEs; IBM). For all tests, *p* values ≤0.05 were considered significant.

## Results

### Effects of intra-articular TA treatment

**Fig. 2:**
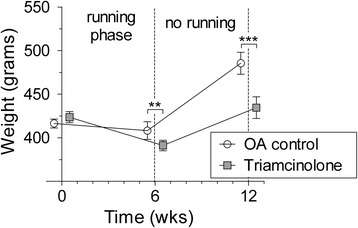
Increase in body weight (in grams) of untreated control rats (*white circles*) and triamcinolone acetonide–treated rats (*grey squares*). *OA* osteoarthritis, **:*p* < 0.01, ***:*p* < 0.001

The mean body weight of all untreated rats at baseline was 416.4 g (411.3–421.5 g), and during 6 weeks of treadmill running this did not increase (mean weight 408.3 g, 398.2–418.3 g). During the subsequent 6 weeks of rest, the body weight of all rats increased (mean 485.5 g, 473.0–498.0 g). TA-treated animals (mean weight at baseline 423.6 g, 417.3–429.9 g) lost body weight during OA induction (mean weight after 6 weeks 391.2 g, 385.1–397.2 g), and they weighed less than untreated OA animals (*p* = 0.004). After 12 weeks, their mean body weight increased to 434.6 g (422.2–446.9 g), but it was still significantly less than that of untreated OA controls (*p* < 0.0001) (Fig. 2).

### Effect of intra-articular TA treatment on synovial macrophage activation

**Fig. 3:**
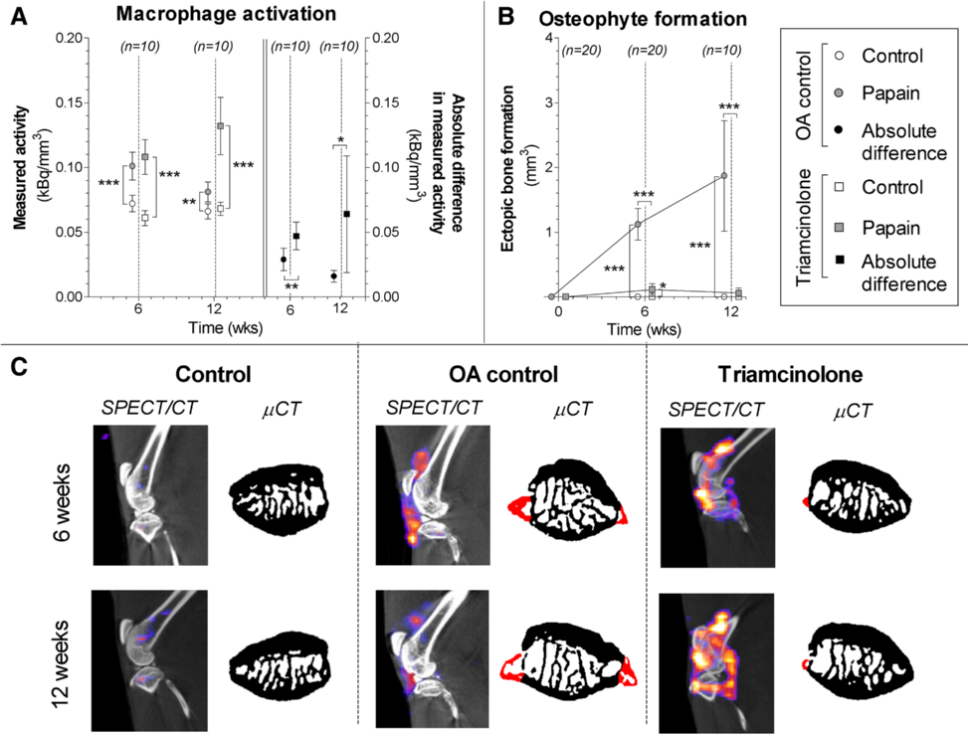
Macrophage activation determined in untreated animals with osteoarthritis (OA) (*circles*) and triamcinolone acetonide–treated animals (*squares*) after injection of ^111^In-EC0800 using single-photon emission computed tomography/computed tomography (SPECT/CT). **a** Quantitative outcome of measured radioactivity in the healthy joints (*blank boxes*) and OA joints (*grey boxes*) normalized to the size of the analyzed cylindrical region of interest (in kilobecquerels per cubic millimetre). Absolute differences per animal were calculated (in kilobecquerels per cubic millimetre) to correct for differences in biodistribution of ^111^In-EC0800 (*black boxes*). High radioactivity is related to more macrophage activation. **b** Ectopic bone formation (in kilobecquerels per cubic millimetre) as a measure for osteophyte development was quantified on longitudinal bone micro–computed tomographic (μCT) scans. **c** Sagittal SPECT/CT images of knee joints from representative animals. μCT images shown in *black* and *white* were used for anatomical reference. The SPECT/CT images are shown in colour. Transaxial images from patellar bone extracted from binary μCT images show ectopic bone formation (*red*). *p < 0.05, **p < 0.01, ***p < 0.001. Error bars indicate 95 % confidence intervals

Each animal received 54 ± 2 MBq of ^111^In-EC0800 under isoflurane anaesthesia, and we observed no significant differences in macrophage activity between experimental groups after injection. Untreated OA control animals showed more macrophage activation in their OA-induced joints at 6 weeks (*p* < 0.0001) and at 12 weeks (*p* < 0.0001). TA-injected knee joints also showed more macrophage activation than their non-injected healthy knee joints (*p* < 0.0001 at 6 and 12 weeks). To correct for differences in biodistribution, we calculated paired absolute differences between healthy control joints and OA-induced joints for all untreated rats and TA-treated animals. At both 6 weeks (*p* = 0.008) and 12 weeks (*p* = 0.04), this analysis suggested more macrophage activation in TA-injected joints (Fig. 3a, c). In line with macrophage activation in untreated animals, OA-induced knee joints showed evident ectopic bone formation in untreated animals compared with their healthy control joints at 6 weeks (*p* < 0.0001) and 12 weeks (*p* < 0.0001). TA-injected joints showed only minimal or no osteophyte formation compared with their healthy control joints (*p* = 0.02 at 6 weeks, *p* = 0.11 at 12 weeks) and compared with untreated OA joints (*p* < 0.0001 at 6 and 12 weeks) (Fig. 3b, c).

### Effect of intra-articular saline injections on macrophage activation

**Fig. 4:**
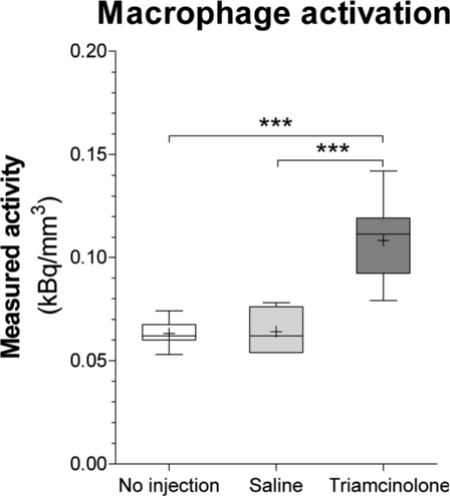
Effect of intra-articular injection on macrophage activation determined with ^111^In-EC0800 single-photon emission computed tomography/computed tomography (SPECT/CT). There was no difference between knee joints without intra-articular injection (*white column*) and knee joints injected with saline (*light grey column*) 1 day before SPECT/CT scanning. Knees injected with triamcinolone acetonide 1 day before SPECT/CT showed clearly increased radioactive uptake, which represents increased macrophage activation. ***:*p* < 0.001

To test whether the amount of macrophage activation in TA-treated animals did not result from the intra-articular injection, we tested in a small experiment whether saline injections also induced macrophage activation. Therefore, we gave five Wistar rats injections with a saline solution into a healthy knee joint. Subsequently, we performed SPECT/CT in these animals using ^111^In-EC0800 as described before. In these animals, we found that there was no difference in measured radioactivity between non-injected and saline-injected knee joints (Fig. 4). This suggests that an intra-articular injection does not induce macrophage activation that explains the additional measured activity in the TA-treated animals in the first experiment.

### Osteoarthritic changes of articular cartilage

**Fig. 5:**
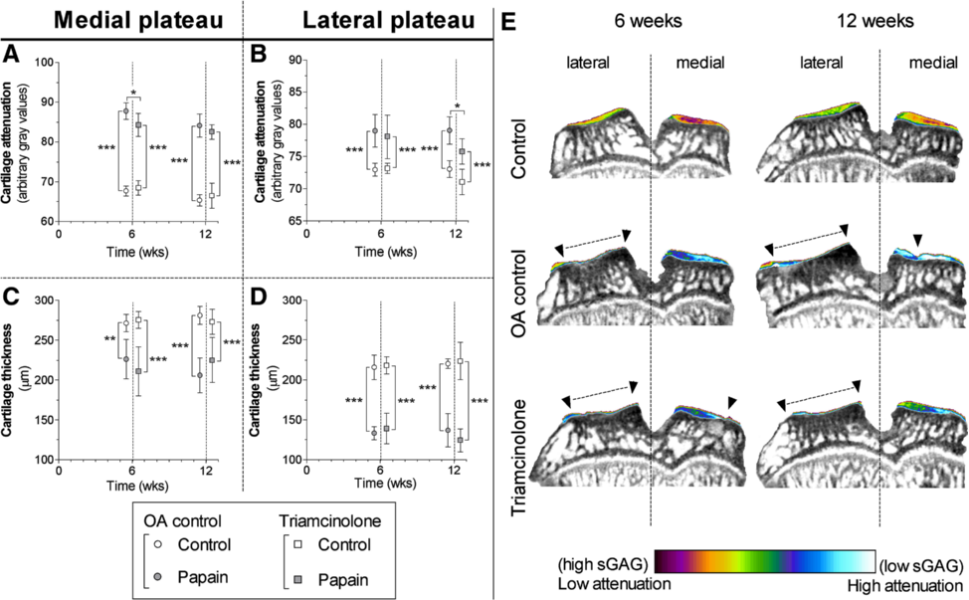
Cartilage quality and quantity were determined in samples of untreated (*circles*) and triamcinolone acetonide–treated (*squares*) rats with equilibrium partitioning of an ionic contrast agent using micro–computed tomography(EPIC-μCT) **a**–**d** The amounts of sulphated glycosaminoglycans (sGAGs) (arbitrary *grey* values in **a** and **b**) and cartilage thickness (in micrometres; **c** and **d**) were measured in the medial (**a** and **c**) and lateral (**b** and **d**) cartilage compartments of the tibial plateau harvested from healthy joints (*open boxes*) and osteoarthritis (OA)-induced joints (*grey boxes*). Attenuation values from EPIC-μCT scans are inversely related to the sGAG content, meaning that a high attenuation corresponds to low sGAG content. Coronal images from representative EPIC-μCT scans of the tibial plateau show the amount of cartilage (erosions indicated with *inverted triangles* and *dashed lines*) and sGAG content (displayed in colour). *p < 0.05, **p < 0.01, ***p < 0.001. Error bars indicate 95 % confidence intervals

**Fig. 6:**
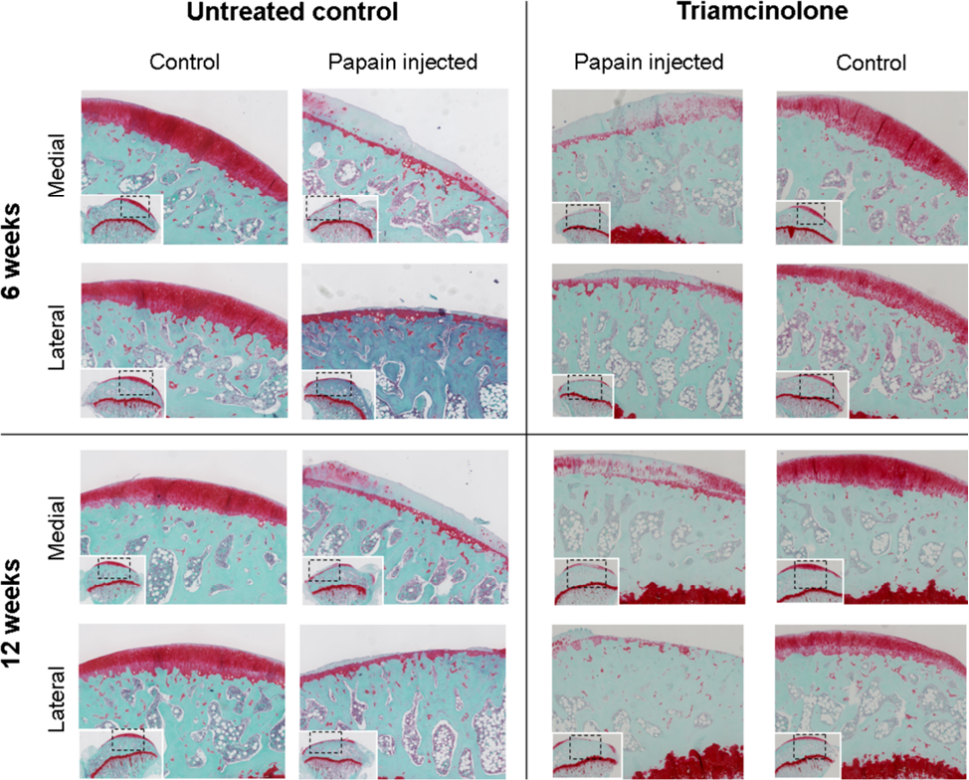
Safranin-O–stained histological sections showing representative images of medial and lateral tibial plateau cartilage after 6 weeks and 12 weeks of follow-up of untreated and triamcinolone acetonide–treated animals. Dashed boxes demonstrate which area was magnified 125x

Both medial and lateral cartilage compartments of the tibial plateau were severely sGAG-depleted in untreated controls at 6 and 12 weeks (Fig. 5a). After the running protocol at 6 weeks, cartilage of the medial compartment was slightly reduced in thickness (Fig. 5c). Lateral cartilage thickness was severely degraded (Fig. 5d) and resulted in almost completely denuded subchondral bone (Fig. 5e). During the subsequent 6 weeks of rest, medial cartilage continued to degrade; in the lateral compartment, an ongoing decline in cartilage thickness was not seen (Fig. 5c–e). sGAG loss and cartilage degradation in TA-treated animals followed the same pattern as in untreated animals. Only at 6 weeks did medial cartilage show slightly decreased attenuation (*p* = 0.04), and at 12 weeks we measured lower attenuation values in lateral cartilage (*p* = 0.02). Figure 6 shows representative medial and lateral cartilage images from Safranin-O–stained histological specimens from untreated controls and TA-treated animals at 6 and 12 weeks.

### Subchondral bone changes

**Fig. 7:**
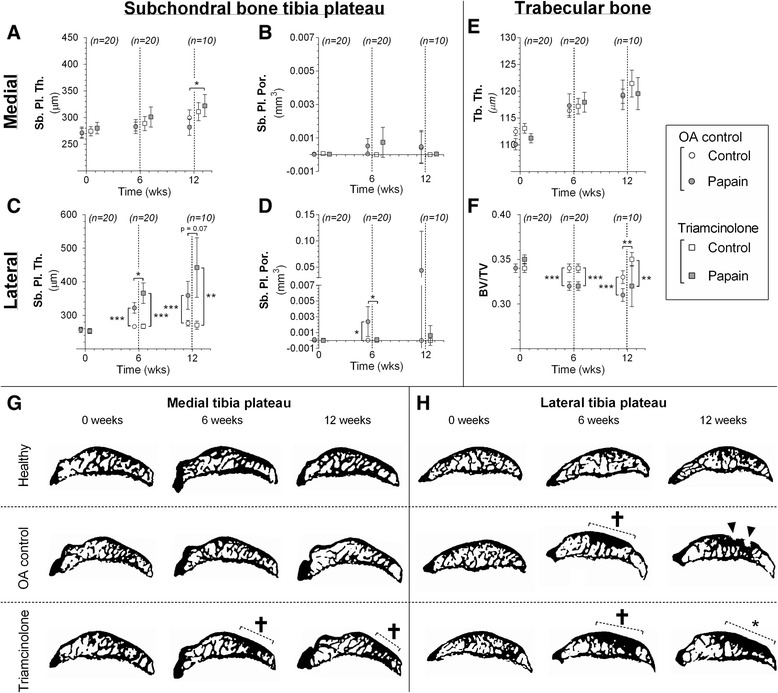
Subchondral bone changes analyzed with longitudinal in vivo micro–computed tomography (μCT) in untreated animals (*circles*) and triamcinolone acetonide–treated animals (*squares*). Subchondral plate thickness (Sb. Pl. Th.; **a** and **c**) and porosity (Sb. Pl. Por.; **b** and **d**) were measured in the medial (**a** and **b**) and lateral (**c** and **d**) compartments of the tibial epiphysis. Changes in trabecular thickness (Tb. Th.; **e**) and trabecular bone volume fraction (BV/TV; **f**) were measured in tibial epiphysis bone marrow. **g** and **h** Representative sagittal images from binary μCT scans show pore development (indicated with *inverted triangles*) and development of subchondral sclerosis (indicated with *dashed lines* and *asterisks*) in the medial (**g**) and lateral (**h**) compartments of the tibial epiphysis. *p < 0.05, **p < 0.01, ***p < 0.001. Error bars indicate 95 % confidence intervals. *OA* osteoarthritis

Compared with the healthy control joints, medial subchondral plates in OA-induced joints of untreated controls tended to decrease in thickness from 6 to 12 weeks but was not significantly different (*p* = 0.16). Medial subchondral plate thickness in TA-treated rats followed more the same pattern as the healthy control joints in these animals (Fig. 7a), but compared with untreated controls the subchondral plate in these animals was slightly thicker at 12 weeks (*p* = 0.01). GEEs showed that medial subchondral bone plates of TA-treated animals were thicker during the experiment than those of untreated controls (*p* = 0.02). Sagittal μCT images showed that this increase in subchondral plate thickness was not generalized as in healthy controls knees, but was more focal and indicative of a sclerotic phenotype (Fig. 7g). Medial plate porosity did not increase in both experimental groups throughout the experiment (Fig. 7b). Lateral compartment subchondral bone thickness of untreated OA joints was clearly increased compared with their healthy control joints at 6 weeks (*p* < 0.0001) and 12 weeks (*p* < 0.0001) (Fig. 7c, h). Longitudinal measured subchondral bone thickness analyzed using GEEs showed that TA-treated animals developed more lateral subchondral sclerosis in their OA-induced joints during the experiment than did those of untreated controls (*p* < 0.0001). Although untreated animals developed minimal subchondral plate porosity at 6 weeks, no differences were found compared with TA-treated animals (Fig. 7d, h).

Trabecular thickness did not differ between experimental groups during the experiment (Fig. 7e). BV/TV ratios were lower in OA-induced joints of both groups than in their healthy knee joints (Fig. 7f), and no differences were found between OA joints of untreated controls or TA-treated animals. However, healthy control joints of TA-treated animals had higher BV/TV ratios than those of healthy control joints of untreated animals (*p* = 0.003).

### Effects of TA treatment on M1 and M2 macrophages cultured in vitro

**Fig. 8:**
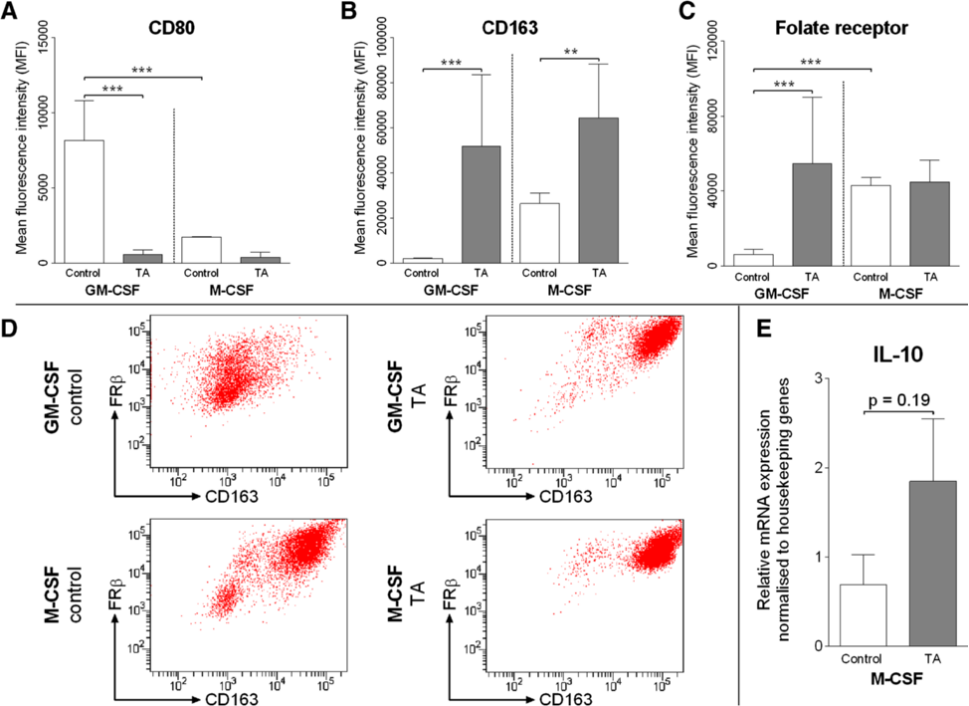
Expression of CD80, CD163 and folate receptor beta (FRβ) analyzed using fluorescence-activated cell sorting in monocyte-derived macrophages with or without triamcinolone acetonide (TA). **a**–**c** Data shown are representative of three independent experiments. **d** Representative flow cytometry plots. **e** Relative *interleukin (IL)-10* messenger RNA expression in monocyte-derived M2 macrophages with or without TA. Data represent two independent experiments.*p < 0.05, **p < 0.01, ***p < 0.001. Error bars indicate standard error of the mean. *GM-CSF* granulocyte-macrophage colony-stimulating factor, *M-CSF* macrophage colony-stimulating factor

Monocyte-derived macrophages differentiated in the presence of GM-CSF showed enhanced expression of CD80, whereas CD163 expression was absent (Fig. 8a, b). When monocytes were exposed to TA in addition to GM-CSF, both CD163 receptor and FRβ expression increased significantly (Fig. 8c). Interestingly, TA strongly decreased survival in GM-CSF–stimulated monocytes, but not in M-CSF–stimulated monocytes. Monocyte-derived macrophages cultured in the presence of M-CSF showed enhanced expression of CD163 and CD16 but absence of CD80 (Fig. 8a, b). FRβ expression in these cells was increased compared with untreated GM-CSF cells, but it was not enhanced by the addition of TA (Fig. 8c). Representative images from fluorescence-activated cell sorting experiments are shown in Fig. 8d. Additionally, TA-treated M-CSF macrophages showed significantly increased levels of *IL10* mRNA expression (Fig. 8e).

## Discussion

In this study, we investigated the effects of TA injections on in vivo macrophage activation during OA progression. In untreated animals, there was a marked increase of activated macrophages measured with in vivo FRβ targeted SPECT/CT imaging (Fig. 3). It is thought that activated macrophages in OA produce TGF-β, which induces BMP production in synoviocytes, subsequently triggering osteophyte development [4, 5]. Therefore, we expected to see progressive growth of patellar osteophytes in untreated animals. In treated animals, however, intra-articular TA injections completely prevented osteophyte development. Interestingly, TA injections severely induced macrophage activation (Fig. 3). Because saline injections did not reproduce this enhanced SPECT/CT signal, we can exclude the injection itself as the cause for macrophage activation (Fig. 4). We hypothesized that this combination of enhanced macrophage activation and prevented osteophytosis might be explained through different subtypes into which macrophages can differentiate.

Therefore, we performed in vitro experiments using GM-CSF– and M-CSF–cultured monocytes. GM-CSF–cultured monocytes were CD80^+^ and CD163^−^, which is typical for classically activated (M1) macrophages [31], whereas M-CSF–cultured monocytes were CD163^+^ and lacked CD80, which is typical for alternatively activated (M2) macrophages [32, 33]. It is known that FRβ is co-expressed predominantly in CD163^+^ macrophages [34]. Our experiments confirm this finding because FRβ was especially elevated in M-CSF–cultured M2 macrophages (Fig. 8c). However, adding TA during GM-CSF drove macrophage differentiation, and these M1-activated macrophages started to co-express FRβ. Interestingly, these cells stop CD80 expression and increase CD163 expression, suggesting that TA stimulates macrophages towards an activated M2 phenotype. Although this TA-induced FRβ^+^ M2-activated phenotype explains the increased SPECT/CT signal in TA-treated animals, it does not explain why TA-treated animals lacked osteophyte formation.

Glucocorticoids are known inducers of a specific macrophage subtype known as regulatory macrophages. These are a specific form of M2-activated macrophage which is considered anti-inflammatory. Through interaction with transcription factors, glucocorticoids regulate macrophage gene expression levels [35]. By induction of inhibitor of nuclear factor κβ, glucocorticoids inhibit nuclear factor κβ [36], which results in decreased production of pro-inflammatory cytokines such as IL-1, IL-6 and TNF [37]. Furthermore, the regulatory macrophage can be characterized by enhanced *IL-10* production [38]. Therefore, we analyzed our M2 macrophages cultured in vitro for mRNA expression of *IL-10*, and we found that TA strongly increased *IL-10* expression levels in M-CSF–cultured monocytes (Fig. 8e). We believe that this underlines that TA strongly polarizes macrophage activation towards a specific anti-inflammatory macrophage subtype that does not promote osteophyte growth in our in vivo model of OA.

Besides the effects of TA on macrophages, we also investigated whether TA could be beneficial for either articular cartilage or subchondral bone. In a previously reported study in which researchers used a pre-clinical animal model of traumatic OA, intra-articular injections with dexamethasone led to less cartilage damage [12]. We could not reproduce this result in our present study. Our cartilage results showed no protection against cartilage erosion of the lateral tibial plateau or against loss of cartilage matrix of the medial plateau (Fig. 5). There were also no beneficial effects of TA against pathological changes within the subchondral bone. In fact, GEE analysis of the medial subchondral plate showed that more subchondral sclerosis developed in TA-treated animals. On the basis of repeated intra-articular injections of TA, it is known that cartilage matrix metabolism is changed, as measured by biomarkers within synovial fluid [39]. Furthermore, corticosteroid treatment is known to induce chondrocyte apoptosis in chondrocyte cultures and in vivo [40]. These data suggest that TA treatment could very well have induced a direct toxic effect for chondrocytes. Subsequently, more chondrocyte death could have enhanced cartilage damage, and therefore more subchondral sclerosis may have developed.

There are several other potential biases that we cannot exclude, owing to our experimental design. First, our animals received 100 μg of TA per injection. This dose was estimated on the basis of interpretation of previously published studies in which authors reported use of corticosteroids in rats and in other species. A frequently mentioned dose is 1 mg/kg body weight, a dose which we further reduced to one-fifth (to 0.2 mg/kg body weight) injected weekly. Because TA injections were done weekly, we cannot exclude possible systemic effects, such as due to TA overdose, that might have influenced macrophage activation or osteophyte formation. An indication for TA overdose in treated animals is suggested through measured weight loss (Fig. 2). However, treated animals still received TA injections after the running protocol, and in this period their weight increased again. We observed that TA-treated animals endured the running protocol better than the untreated controls. It could be that TA reduced pain and improved their running capabilities, which resulted in weight loss. However, we cannot support this hypothesis with further data and are therefore not able to exclude possible toxic effects due to TA treatment. Furthermore, although van Lent et al. [4] and Blom et al. [5] previously underlined the role of macrophage activation in osteophyte formation, we cannot exclude that TA also influenced osteophyte formation directly. For example, TA might have influenced other cell types, such as osteoblasts and osteoclasts, that are also involved during osteophyte formation.

More studies using SPECT/CT imaging techniques are needed to gain more knowledge related to macrophage activation and manipulation through therapeutic strategies in all kinds of disease. Other studies have shown the possibility of visualizing M1 polarization of microglia (a group of macrophages within the brain) in animal models of psychiatric disorders [41]. Different tracers enable differentiation between M1- and M2-activated macrophages [42]. Hopefully, it will be possible in the near future to also use tracers to differentiate between subtypes (e.g., wound-healing and regulatory) of M2 macrophages. These techniques would allow monitoring of specific macrophage subtypes activated in vivo in pre-symptomatic stages of OA and measure effects of pre-emptive intervention strategies dedicated to interfering with macrophage polarization. Eventually, these studies will answer questions about how macrophages and related immune cells might be manipulated more specifically to prevent or delay disease progression.

## Conclusions

Pre-emptive treatment with intra-articular TA injections showed enhanced FRβ-related macrophage activation in an in vivo model of OA and fully prevented osteophyte development. TA strongly induced monocyte differentiation towards an M2 and anti-inflammatory macrophage phenotype. TA leads to increased *IL-10* mRNA levels in vitro and reduced osteophytosis in vivo, which indicates that TA potently induced a CD163^+^ and FRβ^+^ regulatory macrophage. Unfortunately, FRβ cannot be used to differentiate between wound-healing and regulatory M2 subtypes. Future studies should be aimed at identifying specific surface markers for each of these subtypes to enable in vivo identification using imaging techniques such as SPECT/CT. Future fine-tuning of the anti-inflammatory and anti-pain capabilities of M2 subtypes might prove beneficial against disease progression and reduce patient complaints.

## Abbreviations

APC: allophycocyanin
BMP: bone morphogenetic protein
BV: trabecular bone volume
BV/TV: trabecular bone volume fraction
μCT: micro–computed tomography
ECM: extracellular matrix
EPIC-μCT: equilibrium partitioning of an ionic contrast agent using micro–computed tomography
FRβ: folate receptor beta
GEE: generalized estimating equation
GM-CSF: granulocyte-macrophage colony-stimulating factor
IFN-γ: interferon-γ
IL: interleukin
M-CSF: macrophage colony-stimulating factor
mRNA: messenger RNA
OA: osteoarthritis
Sb. Pl. Por.: subchondral plate porosity
Sb. Pl. Th.: subchondral plate thickness
sGAG: sulphated glycosaminoglycan
SPECT/CT: single-photon emission computed tomography/computed tomography
TA: triamcinolone acetonide
Tb. Th.: trabecular thickness
TGF-β: transforming growth factor beta
TNF: tumour necrosis factor

## References

[1] Suri S, Walsh DA (2012). Osteochondral alterations in osteoarthritis. Bone.

[2] Weinans H, Siebelt M, Agricola R, Botter SM, Piscaer TM, Waarsing JH (2012). Pathophysiology of peri-articular bone changes in osteoarthritis. Bone.

[3] Scanzello CR, Goldring SR (2012). The role of synovitis in osteoarthritis pathogenesis. Bone.

[4] van Lent PL, Blom AB, van der Kraan P, Holthuysen AE, Vitters E, van Rooijen N (2004). Crucial role of synovial lining macrophages in the promotion of transforming growth factor β–mediated osteophyte formation. Arthritis Rheum.

[5] Blom AB, van Lent PL, Holthuysen AE, van der Kraan PM, Roth J, van Rooijen N (2004). Synovial lining macrophages mediate osteophyte formation during experimental osteoarthritis. Osteoarthritis Cartilage.

[6] Bondeson J, Blom AB, Wainwright S, Hughes C, Caterson B, van den Berg WB (2010). The role of synovial macrophages and macrophage-produced mediators in driving inflammatory and destructive responses in osteoarthritis. Arthritis Rheum.

[7] de Lange-Brokaar BJ, Ioan-Facsinay A, Yusuf E, Visser AW, Kroon HM, van Osch GJ (2015). Association of pain in knee osteoarthritis with distinct patterns of synovitis. Arthritis Rheumatol.

[8] Bellamy N, Campbell J, Robinson V, Gee T, Bourne R, Wells G (2006). Intraarticular corticosteroid for treatment of osteoarthritis of the knee. Cochrane Database Syst Rev..

[9] McAlindon TE, Bannuru RR, Sullivan MC, Arden NK, Berenbaum F, Bierma-Zeinstra SM (2014). OARSI guidelines for the non-surgical management of knee osteoarthritis. Osteoarthritis Cartilage.

[10] Hepper CT, Halvorson JJ, Duncan ST, Gregory AJ, Dunn WR, Spindler KP (2009). The efficacy and duration of intra-articular corticosteroid injection for knee osteoarthritis: a systematic review of level I studies. J Am Acad Orthop Surg.

[11] Williams JM, Brandt KD (1985). Triamcinolone hexacetonide protects against fibrillation and osteophyte formation following chemically induced articular cartilage damage. Arthritis Rheum.

[12] Huebner KD, Shrive NG, Frank CB (2014). Dexamethasone inhibits inflammation and cartilage damage in a new model of post-traumatic osteoarthritis. J Orthop Res.

[13] Mackaness GB (1977). Cellular immunity and the parasite. Adv Exp Med Biol..

[14] O’Shea JJ, Murray PJ (2008). Cytokine signaling modules in inflammatory responses. Immunity.

[15] Gordon S (2003). Alternative activation of macrophages. Nat Rev.

[16] Loke P, Gallagher I, Nair MG, Zang X, Brombacher F, Mohrs M (2007). Alternative activation is an innate response to injury that requires CD4^+^ T cells to be sustained during chronic infection. J Immunol.

[17] Zizzo G, Hilliard BA, Monestier M, Cohen PL (2012). Efficient clearance of early apoptotic cells by human macrophages requires M2c polarization and MerTK induction. J Immunol.

[18] Martinez FO, Sica A, Mantovani A, Locati M (2008). Macrophage activation and polarization. Front Biosci..

[19] Siebelt M, Groen HC, Koelewijn SJ, de Blois E, Sandker M, Waarsing JH (2014). Increased physical activity severely induces osteoarthritic changes in knee joints with papain induced sulphate-glycosaminoglycan depleted cartilage. Arthritis Res Ther.

[20] Low PS, Henne WA, Doorneweerd DD (2008). Discovery and development of folic-acid-based receptor targeting for imaging and therapy of cancer and inflammatory diseases. Acc Chem Res.

[21] Turk MJ, Breur GJ, Widmer WR, Paulos CM, Xu LC, Grote LA (2002). Folate-targeted imaging of activated macrophages in rats with adjuvant-induced arthritis. Arthritis Rheum.

[22] Murat N, Karadam B, Ozkal S, Karatosun V, Gidener S (2007). [Quantification of papain-induced rat osteoarthritis in relation to time with the Mankin score]. Acta Orthop Traumatol Turc.

[23] Piscaer TM, Müller C, Mindt TL, Lubberts E, Verhaar JA, Krenning EP (2011). Imaging of activated macrophages in experimental osteoarthritis using folate-targeted animal single-photon–emission computed tomography/computed tomography. Arthritis Rheum.

[24] Palmer AW, Guldberg RE, Levenston ME (2006). Analysis of cartilage matrix fixed charge density and three-dimensional morphology via contrast-enhanced microcomputed tomography. Proc Natl Acad Sci U S A.

[25] Waarsing JH, Day JS, Weinans H (2004). An improved segmentation method for in vivo μCT imaging. J Bone Miner Res.

[26] van der Jagt OP, van der Linden JC, Schaden W, van Schie HT, Piscaer TM, Verhaar JA (2009). Unfocused extracorporeal shock wave therapy as potential treatment for osteoporosis. J Orthop Res.

[27] Botter SM, van Osch GJ, Clockaerts S, Waarsing JH, Weinans H, van Leeuwen JP (2011). Osteoarthritis induction leads to early and temporal subchondral plate porosity in the tibial plateau of mice: an in vivo microfocal computed tomography study. Arthritis Rheum.

[28] Xia W, Hilgenbrink AR, Matteson EL, Lockwood MB, Cheng JX, Low PS (2009). A functional folate receptor is induced during macrophage activation and can be used to target drugs to activated macrophages. Blood.

[29] Benito MJ, Veale DJ, FitzGerald O, van den Berg WB, Bresnihan B (2005). Synovial tissue inflammation in early and late osteoarthritis. Ann Rheum Dis.

[30] Silvast TS, Jurvelin JS, Lammi MJ, Töyräs J (2009). pQCT study on diffusion and equilibrium distribution of iodinated anionic contrast agent in human articular cartilage – associations to matrix composition and integrity. Osteoarthritis Cartilage.

[31] Ambarus CA, Krausz S, van Eijk M, Hamann J, Radstake TR, Reedquist KA (2012). Systematic validation of specific phenotypic markers for in vitro polarized human macrophages. J Immunol Methods.

[32] Philippidis P, Mason JC, Evans BJ, Nadra I, Taylor KM, Haskard DO (2004). Hemoglobin scavenger receptor CD163 mediates interleukin-10 release and heme oxygenase-1 synthesis: antiinflammatory monocyte-macrophage responses in vitro, in resolving skin blisters in vivo, and after cardiopulmonary bypass surgery. Circ Res.

[33] Verreck FA, de Boer T, Langenberg DM, van der Zanden L, Ottenhoff TH (2006). Phenotypic and functional profiling of human proinflammatory type-1 and anti-inflammatory type-2 macrophages in response to microbial antigens and IFN-γ- and CD40L-mediated costimulation. J Leukoc Biol.

[34] Nakashima-Matsushita N, Homma T, Yu S, Matsuda T, Sunahara N, Nakamura T (1999). Selective expression of folate receptor β and its possible role in methotrexate transport in synovial macrophages from patients with rheumatoid arthritis. Arthritis Rheum.

[35] Ogawa S, Lozach J, Benner C, Pascual G, Tangirala RK, Westin S (2005). Molecular determinants of crosstalk between nuclear receptors and Toll-like receptors. Cell.

[36] Ghosh S, May MJ, Kopp EB (1998). NF-κB and Rel proteins: evolutionarily conserved mediators of immune responses. Annu Rev Immunol..

[37] Scheinman RI, Gualberto A, Jewell CM, Cidlowski JA, Baldwin AS (1995). Characterization of mechanisms involved in transrepression of NF-κB by activated glucocorticoid receptors. Mol Cell Biol.

[38] Mosser DM, Edwards JP (2008). Exploring the full spectrum of macrophage activation. Nat Rev.

[39] Celeste C, Ionescu M, Robin Poole A, Laverty S (2005). Repeated intraarticular injections of triamcinolone acetonide alter cartilage matrix metabolism measured by biomarkers in synovial fluid. J Orthop Res.

[40] Nakazawa F, Matsuno H, Yudoh K, Watanabe Y, Katayama R, Kimura T (2002). Corticosteroid treatment induces chondrocyte apoptosis in an experimental arthritis model and in chondrocyte cultures. Clin Exp Rheumatol.

[41] Doorduin J, Klein HC, Dierckx RA, James M, Kassiou M, de Vries EF (2009). [^11^C]-DPA-713 and [^18^F]-DPA-714 as new PET tracers for TSPO: a comparison with [^11^C]-(*R*)-PK11195 in a rat model of herpes encephalitis. Mol Imaging Biol.

[42] Van De Wiele C, Sathekge M, Maes A (2014). Targeting monocytes and macrophages by means of SPECT and PET. Q J Nucl Med Mol Imaging.

